# The fecal microbiome and rotavirus vaccine immunogenicity in rural Zimbabwean infants

**DOI:** 10.1016/j.vaccine.2021.07.076

**Published:** 2021-09-07

**Authors:** Ruairi C. Robertson, James A. Church, Thaddeus J. Edens, Kuda Mutasa, Hyun Min Geum, Iman Baharmand, Sandeep K. Gill, Robert Ntozini, Bernard Chasekwa, Lynnea Carr, Florence D. Majo, Beth D. Kirkpatrick, Benjamin Lee, Lawrence H. Moulton, Jean H. Humphrey, Andrew J. Prendergast, Amee R. Manges, the SHINE Trial Team

**Affiliations:** a Centre for Genomics and Child Health, Blizard Institute, Queen Mary University of London, London, UK; b Zvitambo Institute for Maternal and Child Health Research, Harare, Zimbabwe; c Devil’s Staircase Consulting, West Vancouver, British Columbia, Canada; d British Columbia Centre for Disease Control, Vancouver, British Columbia, Canada; e School of Population and Public Health, University of British Columbia, Vancouver, Canada; f Department of Microbiology and Immunology, University of British Columbia, Canada; g Vaccine Testing Center, College of Medicine, University of Vermont, Burlington, VT, USA; h Department of International Health, Johns Hopkins Bloomberg School of Public Health, Baltimore, MD, USA

**Keywords:** Microbiota, Microbiome, Rotavirus, Shotgun metagenomics, Oral rotavirus vaccine

## Abstract

**Background:**

Oral rotavirus vaccine (RVV) immunogenicity is considerably lower in low- versus high-income populations; however, the mechanisms underlying this remain unclear. Previous evidence suggests that the gut microbiota may contribute to differences in oral vaccine efficacy.

**Methods:**

We performed whole metagenome shotgun sequencing on stool samples and measured anti-rotavirus immunoglobulin A in plasma samples from a subset of infants enrolled in a cluster randomized 2 × 2 factorial trial of improved water, sanitation and hygiene and infant feeding in rural Zimbabwe (SHINE trial: NCT01824940). We examined taxonomic microbiome composition and functional metagenome features using random forest models, differential abundance testing and regression analyses to explored associations with RVV immunogenicity.

**Results:**

Among 158 infants with stool samples and anti-rotavirus IgA titres, 34 were RVV seroconverters. The median age at stool collection was 43 days (IQR: 35–68), corresponding to a median of 4 days before the first RVV dose. The infant microbiome was dominated by *Bifidobacterium longum*. The gut microbiome differed significantly between early (≤42 days) and later samples (>42 days) however, we observed no meaningful differences in alpha diversity, beta diversity, species composition or functional metagenomic features by RVV seroconversion status. *Bacteroides thetaiotaomicron* was the only species associated with anti-rotavirus IgA titre. Random forest models poorly classified seroconversion status by both composition and functional microbiome variables.

**Conclusions:**

RVV immunogenicity is low in this rural Zimbabwean setting, however it was not associated with the composition or function of the early-life gut microbiome in this study. Further research is warranted to examine the mechanisms of poor oral RVV efficacy in low-income countries.

## 1 Introduction

Rotavirus is the leading cause of diarrheal morbidity and mortality in children [1]. Although several oral rotavirus vaccines (RVV) are currently available globally, their efficacy varies substantially by population, limiting their impact [2]. Large clinical studies show RVV efficacy of 85–98% against severe rotavirus gastroenteritis in high-income settings [3], [4], compared to only 48% and 39% in South Asia [5] and sub-Saharan Africa [6], respectively. The reasons for poor oral vaccine efficacy in low-income populations remain poorly understood, however intestinal factors may contribute [7], [8], [9].

The gut microbiota plays a critical role in the maturation of early-life immune function and intestinal development [10]. The composition of the early-life gut microbiota may also influence susceptibility to viral infections, since antibiotic treatment and segmented filamentous bacteria both reduce the pathogenicity of rotavirus in mice [11], [12]. Emerging evidence suggests that the early-life gut microbiota influences oral vaccine responses by modulating the immune and metabolic environments of the intestine [13]. In mice, antibiotic treatment impairs vaccine responses, an effect that is reversed following restoration of the microbiota [14]. In adults, narrow-spectrum antibiotics did not alter absolute anti-RV IgA titres following RVV, but did lead to a higher proportion of individuals displaying short-term IgA “boosting” (defined as a ≥ 2-fold rise in anti-RV titre 7 days post-vaccination) compared with placebo or broad-spectrum antibiotics [15]. Observational evidence from children in low-income settings has shown no consistent association between the fecal microbiota and RVV efficacy in India [16], while RVV immunogenicity was associated with fecal microbiota composition in cohorts in Pakistan [17] and Ghana [18]. In these settings, RVV seroconverters compared to non-seroconverters displayed a fecal microbiota more similar to infants from a high-income setting.

We recently reported outcomes of a cluster-randomized trial of improved water, sanitation and hygiene (WASH) in rural Zimbabwe [19]. Among a subset of infants in whom anti-rotavirus IgA titres were measured, only 24% infants seroconverted following oral RVV administration. However, the WASH intervention significantly increased RVV seroconversion by 50% (19.6% in non-WASH arm versus 30.3% in the WASH arm) [20]. We hypothesised that the WASH intervention had reduced the burden of enteropathogens in these infants, thereby improving RVV immunogenicity. However, the WASH intervention had no effect on enteropathogen carriage [21] and enteropathogen burden was not associated with RVV immunogenicity [22]. In this follow-on study, we explore whether alterations in the composition of the fecal microbiome are associated with RVV immunogenicity, by conducting whole metagenome shotgun sequencing of stool samples from infants around the time of RVV. Here we report on (i) the composition and function of the early-life fecal microbiome in this rural LMIC setting and (ii) its association with RVV immunogenicity.

## 2 Materials and methods

### 2.1 Study population

We present a sub-study of the Sanitation Hygiene Infant Nutrition Efficacy (SHINE) trial [19], a 2 × 2 factorial cluster-randomized trial assessing the independent and combined effects of improved WASH and improved infant and young child feeding (IYCF) on stunting and anaemia (NCT01824940). Briefly, 5280 pregnant women in rural Zimbabwe were enrolled and cluster-randomized to standard-of-care, IYCF, WASH or combined IYCF + WASH, and children were followed up until 18 months of age. 1169 HIV-unexposed children were enrolled in a sub-study of the SHINE trial which involved longitudinal specimen collection (including plasma and stool) at 1, 3, 6, 12 and 18 months of age at scheduled study visits in participants’ homes [23]. Sterile collection tubes were provided to mothers, who collected stool samples from their infants on the day of the study visit. Blood samples were collected from infants by trained nurses. Samples were placed in cool boxes immediately upon collection by study nurses and transported by motorbike to field laboratories where they were aliquoted and stored at −80 °C within 6 h of collection, before subsequent transport to the central laboratory in Harare for ongoing storage at −80 °C. Stool samples were subsequently shipped on dry ice from Harare to the British Columbia Centre for Disease Control, Vancouver, Canada to undergo DNA extraction and metagenomics sequencing. A cold chain was maintained throughout and no freeze–thaw cycles occurred between initial freezing and DNA extraction. 158 infants with available plasma samples, oral rotavirus vaccine records and stool samples collected within 30 days of either vaccine dose were selected for analysis in this study. The SHINE trial was approved by the Medical Research Council of Zimbabwe and the Johns Hopkins Bloomberg School of Public Health Committee on Human Research. Written informed consent was obtained from all caregivers before enrolment in the trial.

### 2.2 Sub-study population

For this study, infants were selected from the existing SHINE cohort if they had at least one available plasma sample collected post-rotavirus vaccination and an available stool sample collected at either the 1 or 3-month visits, as per previous analyses [22]. All stool samples were non-diarrheal. We permitted a 30-day window of stool collection pre- or post-vaccination (either RVV dose). This approach was conducted for two reasons: (i) RVV shedding, a proxy for viral vaccine replication, peaks in the first days after either dose [24] but has been recorded up to 84 days post vaccination [25]. Furthermore, IgA typically appears in blood and stool 7–28 days after rotavirus infection or vaccination suggesting that this is a dynamic period of immune induction [26]. We therefore hypothesized that the fecal microbiome soon before, during or after vaccination may influence RVV immunogenicity; (ii) We previously used this 30-day approach to study the association between enteropathogens and RVV immunogenicity [22], thereby allowing us to conduct a consistent analytical approach of the intestinal milieu and RVV immunogenicity. In order to assess the effect of this chosen 30-day time-window around vaccination, we conducted two sensitivity analyses: first, employing a restricted time-window to include those collected prior to 43 days of age (median age of sample collection), all of which were collected prior to first RVV dose; and second, restricting to samples collected within 14-days pre or post-RVV. Infants were excluded if they did not have vaccination data recorded or had not received at least one dose of RVV. If more than one sample was available per child, the stool sample collected closest to the first dose of RVV was chosen for analysis. 67 samples meeting these criteria had metagenomic sequencing data already available as part of a larger analysis of the gut microbiome of infants from the SHINE cohort (unpublished data). A further 91 samples from the SHINE stool biobank meeting the selection criteria for this study were included, leading to a total of 158 participants in the final analysis. This final subset was based on the number of participants with available stool, plasma and RVV immunogenicity data, and is similar to previously published sample sizes [16], [17], [18]. Participants included in this sub-study were representative of the larger SHINE cohort (Table S1). All samples were processed in an identical manner.

Baseline household data and infant morbidity data were recorded by questionnaire and daily morbidity diaries at each visit. Infant diarrhea was defined as any episode of diarrhea recorded prior to RVV dose 1 (either in 7-day recall via questionnaire or in the daily morbidity diary provided to mothers). Data on antibiotic usage was recorded in SHINE study by maternal recall (14-day recall), however this data was considered of low quality, partly due to the potential for mis-identification of medication. Only 5 of 158 participants included in this study recorded antibiotic usage.

### 2.3 Rotavirus vaccination and anti–rotavirus immunoglobulin A assay

Oral monovalent RVV (Rotarix^TM^_;_ GSK Biologicals) was introduced as part of the Zimbabwean Expanded Programme on Immunisation in May 2014 and was administered to infants at 6 and 10 weeks of age concurrently with oral polio vaccine. National vaccination coverage in 2015–2016 was 87–91% [27]. Vaccination was undertaken at local clinics as part of routine national vaccination programmes and was not overseen by the SHINE trial team. Vaccination dates were recorded by SHINE study staff by reviewing child health cards. Plasma anti–rotavirus immunoglobulin A (IgA) was measured on stored plasma samples collected at 1 and 3 months of age by enzyme-linked immunosorbent assay (ELISA) using methods previously described [28]. The primary outcome was RVV seroconversion, defined as a post-vaccine plasma concentration of anti–rotavirus IgA ≥ 20 U/mL in infants who were seronegative (<20 U/mL) pre-vaccination [29]. Secondary outcomes included anti–rotavirus IgA titre and RVV seropositivity, defined as a post-vaccine titre ≥ 20 U/mL, regardless of pre-vaccine titre. All 3 outcomes were considered in participants with at least 1 vaccine dose. We refer to these outcomes collectively as RVV immunogenicity.

### 2.4 Stool metagenome sequencing

DNA was extracted from ~200 mg stool using the Qiagen PowerSoil Kit with bead beating. DNA quality was confirmed by spectrophotometry (SimpliNano) and quantified by fluorometry (Qubit). 1 µg DNA was used as an input for sequencing library preparation following the Illumina TruSeq PCR-free library preparation protocol, using custom end-repair, adenylation and ligation enzyme premixes (New England BioLabs). Constructed libraries were assessed for quality of concentration (qPCR) and size (Tapestation 2200) prior to pooling. 48 barcoded samples were pooled at random in each sequencing run. Positive controls (ZymoBIOMICS) and DNA-free negative controls were included through all steps including DNA extraction and library preparation. One negative control was included in each sequencing pool. Duplicate samples were also run across different sequencing pools to monitor sequencing batch effects. Whole metagenome sequencing was performed with 125-nucleotide paired-end reads using either the Illumina HiSeq 2500 or HiSeqX platforms at Canada’s Michael Smith Genome Sciences Centre, Vancouver, Canada.

*KneadData* was used with default settings to remove short reads (<60 bp), duplicate reads, human and other non-microbial reads and to trim off adapters. Filtered sequencing reads were processed through the *MetaPhlAn3* pipeline [30] with default settings to generate compositional data. *HUMAnN3* was used with default settings using the UniRef90 database to generate functional annotations (enzyme commission (EC) annotations and pathways) [31]. Compositional and functional data were filtered using a minimum threshold of > 0.1% and > 0.0001% relative abundance, respectively. Taxa were included in the analyses if present at a minimum threshold of 5% prevalence within the entire dataset.

A median of 8.1 ± 3.0 million quality filtered reads were produced per sample. Sixteen negative controls were also sequenced with mean 734 quality-filtered reads (range = 149 to 11,432, SD = 3462). Following filtering of annotatable reads, 139 species were included in the final analysis, of which 4 were classified as co-abundance gene groups metagenomic assemblies (CAGs) for which no culture-derived representative exists.

### 2.5 Statistical analyses

All data were analysed using R (v.4.0.2). Beta-diversity was estimated using the Bray-Curtis dissimilarity index. Alpha diversity was assessed using the Shannon index and the number of observed species. Differential abundance analysis was performed by Wilcoxon rank sum test and Analysis of Composition of Microbiomes (*ANCOM*) [32]. Random forest machine learning models were performed using the *randomForest* package (*SIAMCAT* package [33]) in which five-fold cross-validation was performed with 100 iterations. Microbiome regression analyses were performed using the *MaAsLin2* package. Seven covariates were chosen for adjustment in regression models and included age at stool sample collection, birthweight, exclusive breastfeeding status (recorded at 3 months old), sex, WASH randomized trial arm, delivery mode and length-for-age Z-score (LAZ) around time of vaccination. These covariates were chosen based on biological plausibility and previous evidence of their independent influence on RVV immunogenicity in the same cohort [34]. Multiple comparisons were tested using Benjamini-Hochberg false discovery rate (FDR; q-value < 0.05).

## 3 Results

### 3.1 Study cohort

**Fig 1:**
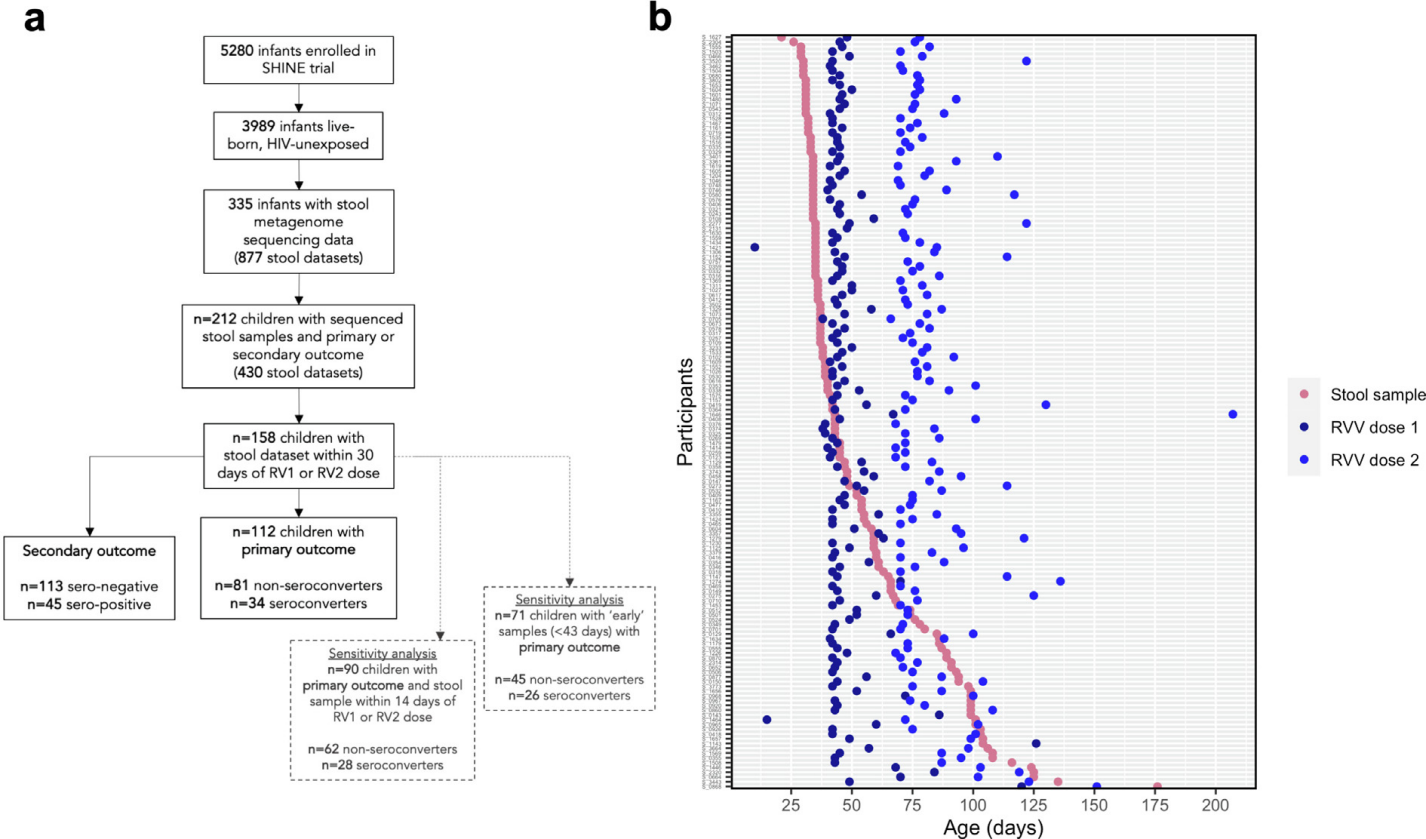
Study flow. (a) Flow diagram of stool samples included in the analysis originating from the SHINE trial. (b) Timing of stool samples included in the analysis and associated RVV doses.

Among 5280 women enrolled in the SHINE trial, there were 3989 live-born, HIV-unexposed infants. Of these, 882 children had RVV immunogenicity measured and 158 of these with stool samples collected within 30 days of either vaccine dose underwent whole metagenome sequencing. Table 1 outlines the primary characteristics of the 158 participants in this analysis. Of these 158 participants, 115 had seroconversion data available as a primary outcome (n = 81 non-seroconverters, n = 34 seroconverters; Fig. 1**a**), whilst secondary outcomes (seropositivity status and IgA titres) were available for all 158 infants (n = 113 seronegative, n = 45 seropositive). 154 of 158 (97.5%) infants had documented receipt of both RVV doses. Seroconverters received their first dose a median of 2 days (P = 0.002) and their second dose a median of 4.5 days (P = 0.037) prior to non-seroconverters. 15/34 (44.1%) of seroconverters and 31/81 (38.3%) of non-seroconverters were female (P > 0.05). The baseline characteristics of the 158 included infants were largely representative of the larger SHINE cohort (Table S1).

**Table 1:** Baseline characteristics of the seroconversion and seropositivity cohorts

	Seroconversion			Seropositivity		
	Non-seroconverters	Seroconverters	P value	Seronegative	Seropositive	P value
	N = 81	N = 34		N = 113	N = 45	
**Female, %**	38.3	44.1		40.7	51.1	
**Birthweight, kg (sd)**	3.17 (0.48)	3.06 (0.55)	0.303	3.11 (0.49)	3.06 (0.50)	0.564
**Low birthweight**^1^**, %**	4.94	11.8		7.96	8.89	
**Vaginal delivery, %**	96.3	97.1		95.6	95.6	
**Institutional delivery, %**	91.2	94.1		93.8	95.6	
**Exclusively breastfed (at month 3), %**	90	97.1		90.1	95.5	
**Concurent OPV vaccination, %**	95.7	93.8		96.5	92.1	
**Age at stool sample, days [IQR]**	38.0 [34.0; 66.0]	37.0 [33.2;40.8]	0.222	45.0 [35.0; 78.0]	38.0 [34.0; 59.0]	0.034
**Age at first RV dose, days [IQR]**	46.0 [43.0; 54.0]	44.0 [42.0;45.0]	0.002	45.0 [42.0; 50.0]	44.0 [42.0; 45.0]	0.019
**Age at second RV dose, days [IQR]**	81.5 [74.2; 99.5]	77.0 [75.0;81.8]	0.037	78.0 [72.0; 93.0]	77.0 [74.8; 83.2]	0.669
**LAZ at 1 month visit [IQR]**	−0.88 (1.09)	−0.80 (1.43)	0.783	−1.00 (1.19)	−0.73 (1.33)	0.267
**LAZ at 3 month visit (sd)**	−0.90 (1.07)	−0.84 (1.19)	0.786	−0.94 (1.13)	−0.88 (1.25)	0.791
**SHINE trial arm**^2^**, %**						
***SOC***	29.6	17.6		30.1	17.8	
***IYCF***	28.4	35.3		28.3	31.1	
***WASH***	19.8	17.6		21.2	20	
***WASH + IYCF***	22.2	29.4		20.4	31.1	
**Born in RV season**^3^**, %**	33.3	44.1		29.2	37.8	
**Household size**	5 [4; 6]	5 [4; 6]	0.126	5.00 [4; 6]	5.00 [4; 6]	0.298
**Mother age at baseline, years (sd)**	27.9 (5.77)	27.3 (6.09)	0.659	27.6 (5.96)	27.2 (6.50)	0.787
**Mother height at baseline, cm (sd)**	161 (6.17)	161 (5.95)	0.983	160 (5.90)	161 (6.24)	0.722
**Both RVV doses received, %**	96.3	100		97.3	97.8	
**Diarrhea pre-RVV**^4^**, %**	11.2	11.8		10.7	11.1	

1 Low birthweight calculated as < 2500 g.

2 SOC, Standard of Care; IYCF, Infant and Young Child Feeding; WASH, Water, Sanitation and Hygeine; WASH + IYCF, combined WASH and IYCF arm.

3 RV season in Zimbabwe was defined as 1st April-31st July based on national surveillance data.

4 Defined as any episode of diarrhea recorded pre-RVV (either in 7-day recall via questionnaire or in the daily morbidity diary provided to mothers).

The median age at RVV was 44 days [interquartile range (IQR): 42–49] for the first dose and 77 days (IQR: 72–88) for the second dose. The median age at pre-vaccine RV IgA titre measurement was 37 days (IQR: 34–45) and 102 days (IQR: 98–116) for post-vaccine titre. The median age at stool sample collection was 43 days (IQR: 35–68), corresponding to a median of 4 days (IQR: +11 to −22) before the first RVV dose (Fig. 1**b**). To test for and account for age-associated changes in microbiota composition, analyses were also split by early stool samples (≤42 days of age), all of which were collected prior to the first RVV dose, and late stool samples (>42 days of age).

### 3.2 Microbiome alpha and beta diversity associations with RVV immunogenicity

**Fig. 2:**
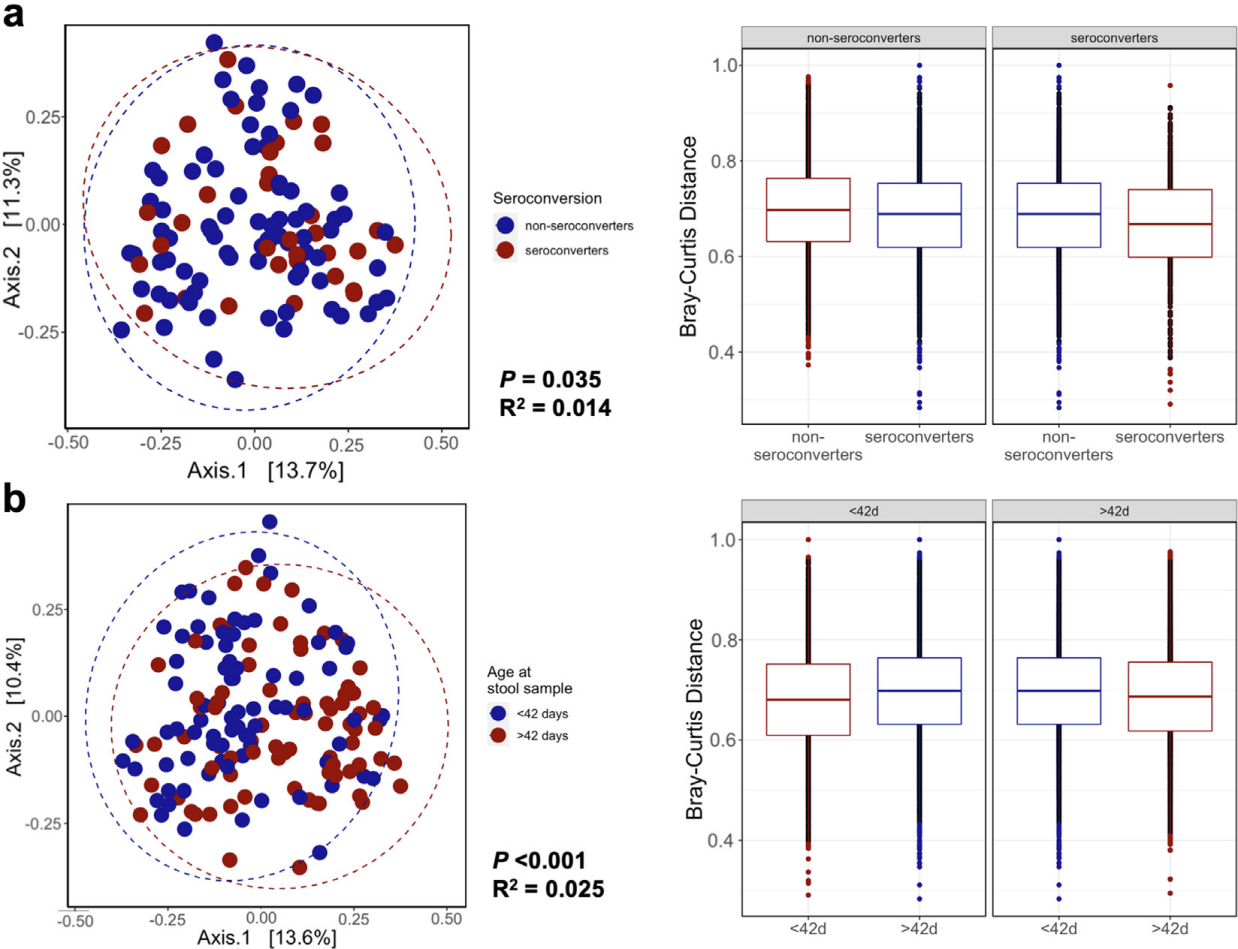
Beta diversity analysis by seroconversion status and early vs late samples. Principal coordinates analysis (PCoA) and associated Bray-Curtis distances to assess beta diversity of seroconversion status (a) and early vs late samples (b) as assessed using PERMANOVA analysis.

PERMANOVA analysis identified a small but significant difference in beta diversity as measured by Bray-Curtis distances by seroconversion status (P = 0.035; R^2^ = 0.014; Fig. 2**a**), however this was largely explained by data dispersion (homogeneity of dispersion test; P = 0.035). When split by median age of sample collection (43 days), there was a significant difference in Bray-Curtis distances between early and late samples (P < 0.001; R^2^ = 0.025; Fig. 2**b**). No significant difference was observed between infants randomized to WASH or non-WASH trial arms (Fig. S1**a).**

In sensitivity analyses, when restricting to samples collected within 14 days of either vaccine dose (n = 62 non-seroconverters, n = 28 seroconverters), there was a significant difference in Bray-Curtis distances by seroconversion status (P = 0.011; R^2^ = 0.022; Fig. S1**b**), which was partly explained by data dispersion (P = 0.03). However, there was no significant difference in Bray-Curtis distances by seroconversion status when restricted to early samples (≤42 days of age; n = 79 samples; P = 0.061; R^2^ = 0.024). In analyses of secondary outcomes, beta diversity differed significantly by seropositivity status (P = 0.008; R^2^ = 0.012; Fig. S1**c**) in the total dataset, which was not explained by dispersion (P = 0.067), but not when restricted to samples collected before ≤ 42 days of age (P = 0.113; R^2^ = 0.019).

**Fig. 3:**
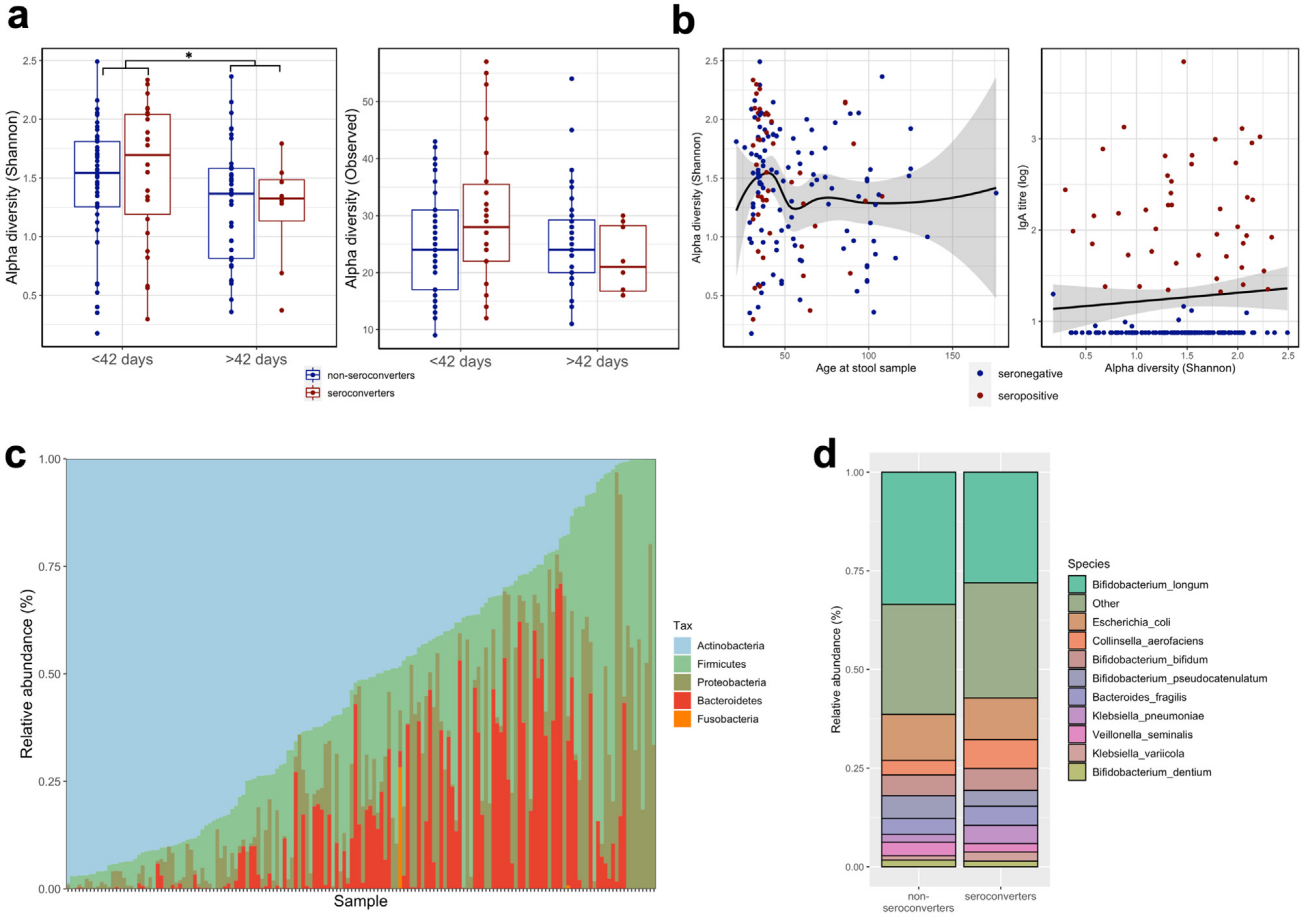
Alpha diversity and taxonomic composition. (a) Alpha diversity, as assessed using the Shannon index and number of observed species, between serconverters and non-seroconverters. (b) Associations between alpha diversity, age at stool sample collection and RV IgA titre. (c) Phylum composition of entire dataset. (d) Species composition in seroconverters vs non-seroconverters.

Alpha diversity, as measured by the Shannon index and number of observed species, did not significantly differ by seroconversion (Wilcoxon test: P = 0.366 & P = 0.282 respectively; Fig. 3**a**) and was not associated with anti-rotavirus IgA titre in simple linear regression (*P* = 0.339; Fig. 3**b**). There were significant differences in alpha diversity between early and late samples (P = 0.008).

In sensitivity analyses, alpha diversity also did not differ by seroconversion status or seropositivity status when restricted to early samples (P = 0.327 and P = 0.269, respectively) or when restricted to stool samples collected within 14 days of either RVV dose (data not shown). There was also no significant difference in alpha diversity by seropositivity status (P = 0.153; Fig. S2**a-b**) or by randomized WASH arms (Fig. S2**c)**.

### 3.3 Infant microbiome taxonomic composition and association with RVV immunogenicity

**Fig. 4:**
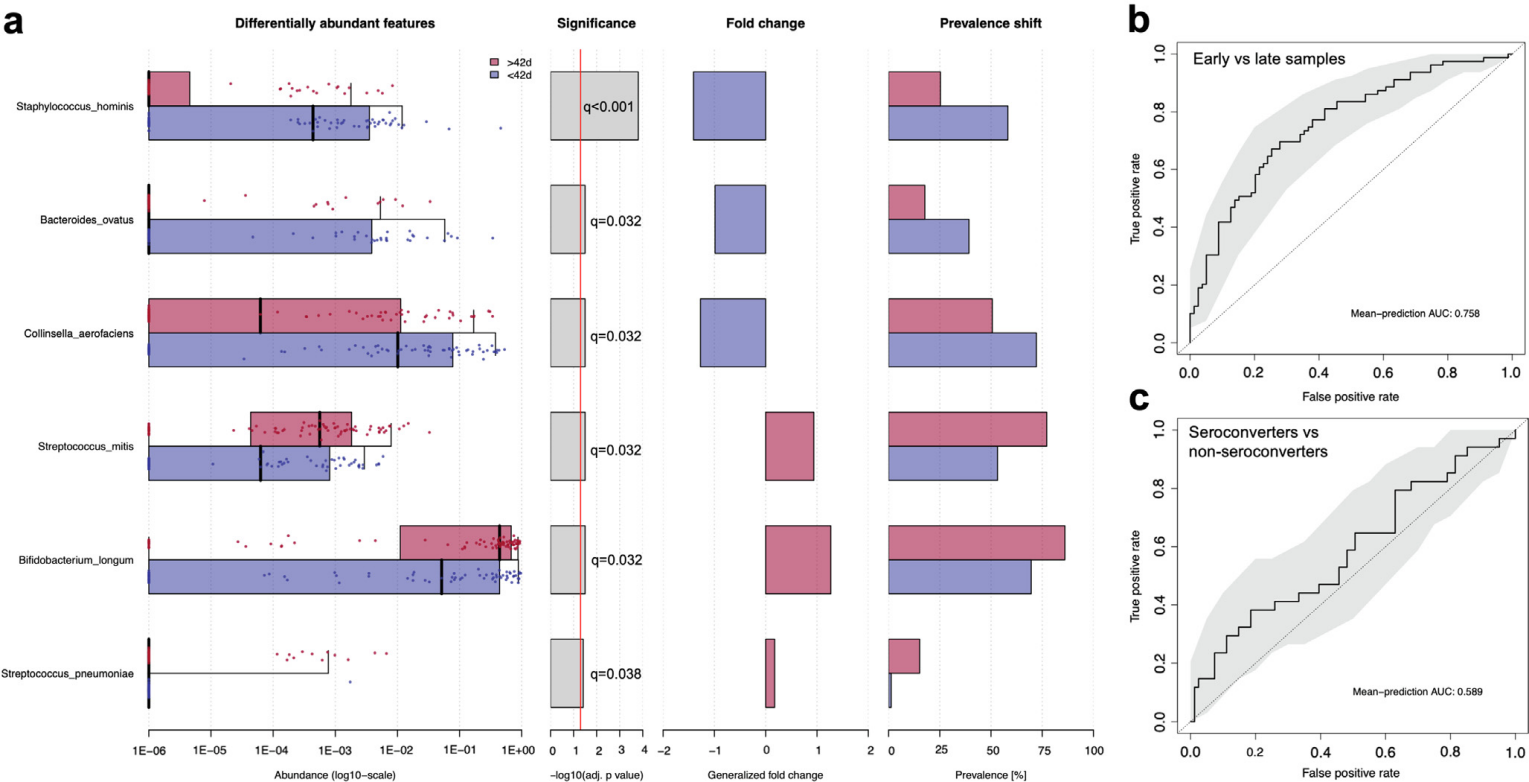
Differential abundance analysis and machine learning models of taxonomic composition. (a) Species that differ significantly in relative abundance between early (≤42 days of age) and late (>42 days of age) samples as assessed by Wilcoxon ranked sum test (q value < 0.05). Random forest machine learning classification model of (a) early vs late samples and (b) seroconverters vs non-seroconverters using all microbiome species.

At the phylum level, samples were dominated by Actinobacteria (Fig. 3**c)**. *Bifidobacterium longum* was the most abundant species in the entire dataset comprising a median 23% relative abundance, ranging from 0% to 97% across all samples. *Escherichia coli, Collinsella aerofaciens, B ifidobacterium bifidum* and *Bifidobacterium pseudocatenulanum* were the next most highly abundant across all datasets. Differential abundance analysis showed no significant differences in relative abundances of any taxa by seroconversion status (Wilcoxon ranked sum test, q > 0.05; Fig. 3**d**). This negative result was confirmed by the more stringent ANCOM model, which included covariate adjustment for age at stool sample collection, birthweight, exclusive breastfeeding status, sex, trial arm, delivery mode and LAZ around vaccination. Six species were significantly differentially abundant between early and late samples (Wilcoxon, q < 0.05: *B. longum*, *Streptococcus mitis, Bacteroides ovatus, Collinsella aerofaciens, Staphylococcus hominis, Streptococcus pneumoniae*; Fig. 4**a and S2e**) which was confirmed by ANCOM following covariate adjustment. In analysis of secondary outcomes, no taxa were significantly different by seropositivity status (Fig. S2**f**). In sensitivity analyses, when restricted to early samples or restricted to samples collected within 14 days of either vaccine dose, no taxa were significantly differentially abundant when compared by seroconversion or seropositivity statuses. *Collinsella aerofaciens* was the only species significantly different in relative abundance between WASH and non-WASH infants in this sub-cohort, displaying significantly lower relative abundance in WASH infants (Fig. S2**g**).

Random forest models were also applied to the datasets to test whether microbiome features could predict seroconversion and age. Early and late samples were strongly classified by the random forest model (receiver operating curve area under the curve (AUC) 0.758; Fig. 4**b**). Random forest regression of age produced moderately strong models (*P* = 0.001; R^2^ = 0.152; 14% explained variation). However, random forest poorly classified seroconversion status (AUC 0.589; Fig. 4**c**). In analysis of other secondary outcomes, random forest also poorly classified seropositivity (AUC 0.564; Fig. S3**a**) and WASH trial arm (AUC 0.5; Fig. S3**b**).

Regression models were built (MaAsLin2) to identify associations between fecal microbial taxa and age or vaccine titres. Following FDR correction and adjustment for covariates, *Bacteroides thetaiotaomicron* was the only species associated with IgA titre, displaying a significant positive correlation (Fig. S4a; q = 0.008). This effect held in sensitivity analyses using samples collected within 14 days of either vaccine dose (q = 0.053) whilst also indicating that *Slackia isoflavoniconvertens* was also positively associated with IgA titre in the restricted sensitivity dataset (Fig. S4b; q = 0.049).

### 3.4 Infant microbiome functional composition and association with RVV immunogenicity

**Fig. 5:**
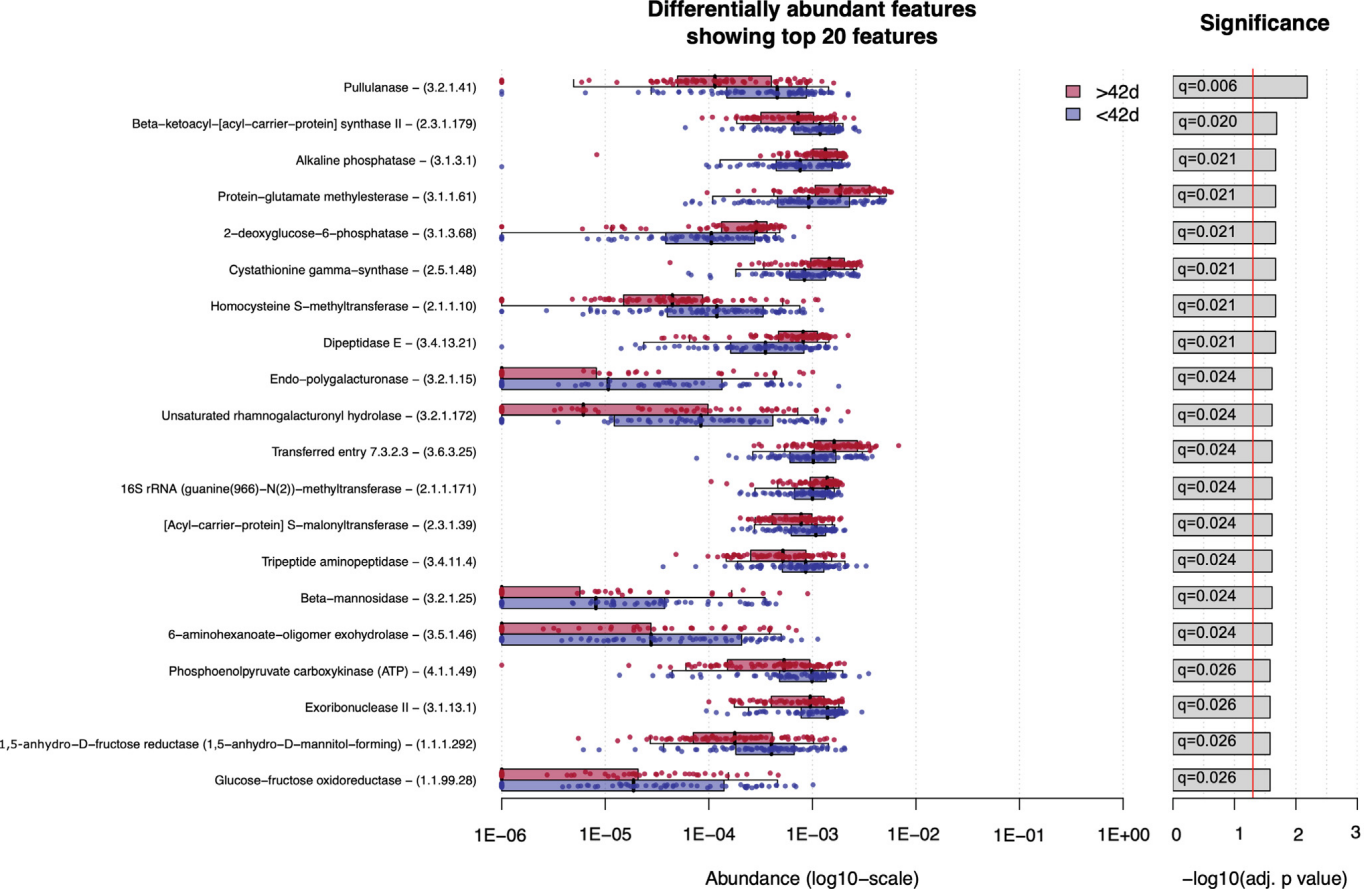
Functional metagenomic analysis. Enzyme commission annotations that differ significantly in relative abundance between early vs late samples (Wilcoxon rank sum test; q value < 0.05; top 20 ECs shown as ranked by q-value).

Metagenomic data were also analysed to assess microbiome functional pathways associated with age and RVV responses. Wilcoxon tests identified no functional pathway or EC that differed significantly by seroconversion status. Random forest models of microbiome functional pathways also poorly classified seroconversion status. Two metagenomic pathways (*PWY − 5994: palmitate biosynthesis I (animals and fungi* and *PWY − 1042: glycolysis IV (plant cytosol)*) and 153 ECs were differentially abundant between early and late samples (Fig. 5). Random forest models performed moderately well in classifying early and late samples based on metagenomic pathways (AUC 0.641; Fig. S3**c**). No EC or metagenomic pathway was significantly associated with IgA titre. In sensitivity analyses of early samples or those collected within 14 days of either vaccine dose, no EC or metagenomic pathway was associated with seroconversion and random forest classification was poor. No significant functional differences were observed by seropositivity status.

## 4 Discussion

Here, we report on the stool metagenomes of 158 infants < 6 months of age from a rural Zimbabwean birth cohort and their association with RVV immunogenicity. Similar to other low-income settings, RVV was poorly immunogenic in this cohort (24% seroconversion). The reasons for this are unclear, but dynamic changes in the microbiota at this age may influence mucosal immune responses to an oral vaccine. However, we do not find distinct fecal microbiome signatures that distinguish RVV seroconverters from non-seroconverters. Hence, poor vaccine seroconversion in this population does not appear to be explained by the fecal microbiome.

A handful of previous studies have demonstrated conflicting evidence for the association between the infant fecal microbiome and oral RVV efficacy. Most recently, a large multi-country study reported that gut microbiota alpha diversity, as assessed using 16S amplicon sequencing, was negatively correlated with RVV immunogenicity in Malawi and India, however no differences were observed in taxonomic composition between seroconverters and non-seroconverters [35]. A previous analysis of 170 infants in south India failed to find an association between the commensal infant fecal microbiome and RVV seroconversion [16]. In the same study, however, RVV seroconverters were more likely to harbour at least one bacterial enteropathogen than non-seroconverters. Conversely, two separate studies in Ghana (n = 20) [18] and Pakistan (n = 78) [17], employing HITChip microarrays rather than next-generation sequencing, both demonstrated that the fecal microbiome of RVV seroconverters differed significantly to non-seroconverters. Both of these studies also reported that the fecal microbiome of seroconverters versus non-seroconverters were more similar to infants from a high-income setting in the Netherlands. However, the taxa associated with RVV seroconversion differed between countries. Higher relative abundances of Clostridium cluster XI and Proteobacteria were associated with seroconversion in Pakistan, whilst higher *Streptococcus bovis* and lower Bacteroidetes were associated with seroconversion in Ghana.

Due to the methodological differences in sequencing and the geographic differences in the cohorts, it is difficult to draw comparisons with these previous studies. Geographic setting has a major influence on gut microbiome composition and inter-individual variation between children across geographical settings is greater than in adults [36]. The results presented here, using gold-standard metagenome sequencing, failed to find a clear infant microbiome signature that distinguished RVV seroconverters from non-seroconverters. Previous evidence shows that the strongest contributor to infant gut microbiome composition in early-life is exclusivity of breastfeeding [37], which was extremely high in this cohort (>80%) due to study interventions designed to promote early and exclusive breastfeeding. Hence, any gut microbiome signatures associated with RVV seroconversion may be outweighed by the influence of breastfeeding. Other environmental factors in early-life can influence gut microbiome composition, including delivery mode, malnutrition and sex. An in-depth analysis of the influence of each of these environmental factors on gut microbiome composition in this cohort was beyond the scope of this current study. However these variables were all included as covariates in each of the models reported here, suggesting that the null association between the gut microbiome and oral rotavirus vaccine efficacy reported here was not influenced by any of these potential confounders.

We recently reported that enteropathogen carriage was not associated with RVV seroconversion in this cohort [22]. The results presented here extend these observations by showing that neither commensal nor pathogenic enteric microbes were associated with RVV seroconversion. Oral RVV is taken up in the small intestine, whereas the microbiome and enteropathogen carriage measured in stool largely reflect the colonic intestinal environment. This may partly explain why we observed no association between the stool microbiota and RVV seroconversion in this cohort. Future studies examining the small intestinal microbiota may provide greater insight into the potential interaction between the commensal gut microbiome and oral vaccines. It remains unclear what biological factors contribute to poor RVV immunogenicity in low-income settings. Improved WASH enhanced RVV immunogenicity by 50% in this cohort [20], yet had no impact on enteropathogen carriage [21] or diarrhea [19]. In this subset of infants, WASH similarly had no significant impact on the early-life fecal microbiome, suggesting that the effect of WASH on RVV immunogenicity is not driven through the fecal microbiome. Hence, the causal pathway linking improved WASH with improved RVV seroconversion remains unexplained and complex.

This study benefited from a well-characterized cohort of infants from a large, community-based, cluster randomized trial. RVV seroconversion, measured in a subset of infants, was 23.7%, demonstrating its suitability as a representative infant population with low RVV immunogenicity. Within this subgroup, we have previously investigated and reported on a number of environmental factors associated with RVV seroconversion including enteropathogen carriage, WASH, household factors, birthweight and nutritional status [20], [22], [34]. Hence the data presented here, albeit negative, add to the evidence base of RVV immunogenicity in a unique, well-characterised cohort by contributing unique insight into the intestinal milieu and its association with oral vaccine efficacy. We employed whole metagenome shotgun sequencing, which has not previously been extensively conducted in low-income settings, allowing us to examine both taxonomic and functional microbiome variables in relation to RVV seroconversion. It is also one of the very few high-resolution, metagenomic datasets from infants in a rural, non-industrialized setting.

The study is limited by its relatively small sample size, which is partly due to the low seroconversion rate in this population, the number of stool samples available for sequencing analysis and the criteria of sample selection within a 30-day time-window close to vaccination. This arbitrary time-window was based on the hypothesis that the intestinal environment around the time of vaccination may alter vaccine efficacy. RVV shedding, a proxy for viral vaccine replication, peaks in the first days after either dose [24] but has been recorded up to 84 days post vaccination [25]. Furthermore, IgA typically appears in blood and stool 7–28 days after rotavirus infection or vaccination, suggesting that this is a dynamic period of immune induction [26]. We therefore hypothesized that the infant gut microbiome in the days/weeks immediately before or after vaccination, during viral vaccine replication, may influence subsequent immunogenicity. Furthermore, this approach complemented our previous analyses of enteropathogens and RVV immunogenicity in this same cohort using the same cut-off around vaccination, thereby providing us with a consistent analytical approach with which to investigate the intestinal milieu and RVV immunogenicity [22].

The study is limited, however, by this sampling approach. The relatively wide dispersion of ages and time-windows between vaccination and stool sample collection may lead to confounding effects of age on any potential gut microbiome associations, which must be considered in the interpretation of these results. To mitigate this, we adjusted for age in all regression models and also performed sensitivity analyses using a restricted 14-day time window and analysing a subset of ‘early’ samples collected before 43 days of age (median age of sample set), all of which were collected prior to vaccination. Sensitivity analyses using both of these restricted datasets also did not identify any comprehensive associations between the fecal microbiota and RVV immunogenicity, whereby no differences were observed in alpha diversity or the relative abundance of any individual species, gene or metagenomic pathway between seroconverters and non-seroconverters. Despite these approaches to account for the dispersion of ages and times between stool samples and vaccination, this analytical approach may still limit our ability to identify true associations between the gut microbiome and RVV seroconversion.

Secondly, the outcomes assessed in this study (seroconversion and seropositivity) may not be accurate correlates of vaccine protection against rotavirus infection [38]. We focussed on seroconversion as a primary outcome as this depends on both pre and post-vaccine titres and also included seropositivity as a secondary analysis. However, as we and others have reported [34], natural rotavirus infection prior to vaccination may confound IgA titres which limits both seroconversion and seropositivity in particular as an outcome. Hence, RV IgA titres are an imperfect correlate of vaccine protection and future studies incorporating other correlates, such as vaccine virus replication, are warranted. Thirdly, the SHINE study was not a vaccine trial and hence the study team had no control over the administration, dosing or timing of vaccinations, which were conducted independently of the SHINE trial as part of the Zimbabwean Expanded Programme on Immunisation. We relied on vaccine data transcribed from participant health cards. The timing of stool collection, and therefore microbiome assessment, was independent of vaccine administration. Additionally, 4/158 (2.5%) participants had documented receipt of only 1 RVV dose. However, we have previously reported in the SHINE study that seroconversion rates in those reporting ≥ 1-dose of RVV were similar to those with 2 reported RVV doses, and the beneficial effects of WASH on these seroconversion rates were the same regardless of 1 or 2 doses [20], [34]. Finally, due to the difficulties in obtaining viral reads from stool metagenomic data, we were unable to determine the impact of enteroviruses on RVV seroconversion, which has been hypothesized previously to be associated with RVV immunogenicity [16]. Specific analysis of the infant virome may shed more light on RVV underperformance.

In conclusion, we found no clear stool microbiome signature associated with oral RVV immunogenicity in rural Zimbabwean infants. Future studies incorporating protection from rotavirus disease, are warranted.

## Funding

This work was supported by the Wellcome Trust [203905/Z/16/Z to JAC, 206455/Z/17/Z to RCR and 093768/Z/10/Z and 108065/Z/15/Z to AJP]. The SHINE trial was funded by the Bill and Melinda Gates Foundation [OPP1021542 and OPP113707]; UK Department for International Development (UK Aid); Swiss Agency for Development and Cooperation and US National Institutes of Health [2R01HD060338-06]. The study funders approved the trial design, but were not involved in data collection, analysis, interpretation, or manuscript preparation.

## Data sharing statement

All relevant data are within the paper and its Supporting Information files except for the raw data which the trial team will begin loading as individual participant data with an accompanying data dictionary at http://ClinEpiDB.org. This platform is charged with ensuring that epidemiological studies are fully anonymized by removing all personal identifiers and obfuscating all dates per participant through application of a random number algorithm to comply with the ethical conduct of human subjects research. Researchers must agree to the policies and comply with the mechanism of ClinEpiDB to access data housed on this platform. Prior to that time, the data are housed on the ClinEpiDB platform at the Zvitambo Institute for Maternal and Child Health Research and available upon request from Ms. Virginia Sauramba (vsauramba@zvitambo.co. zw).

## Declaration of Competing Interest

The authors declare that they have no known competing financial interests or personal relationships that could have appeared to influence the work reported in this paper.
