# Supplementary material for: The fecal microbiome and rotavirus vaccine immunogenicity in rural Zimbabwean infants
Source: Vaccine. 2021 Sep 7;39(38):5391–400. doi: 10.1016/j.vaccine.2021.07.076 (PMC8423000; doi:10.1016/j.vaccine.2021.07.076)
Supplement: Supplementary data 1 [file mmc1.docx]

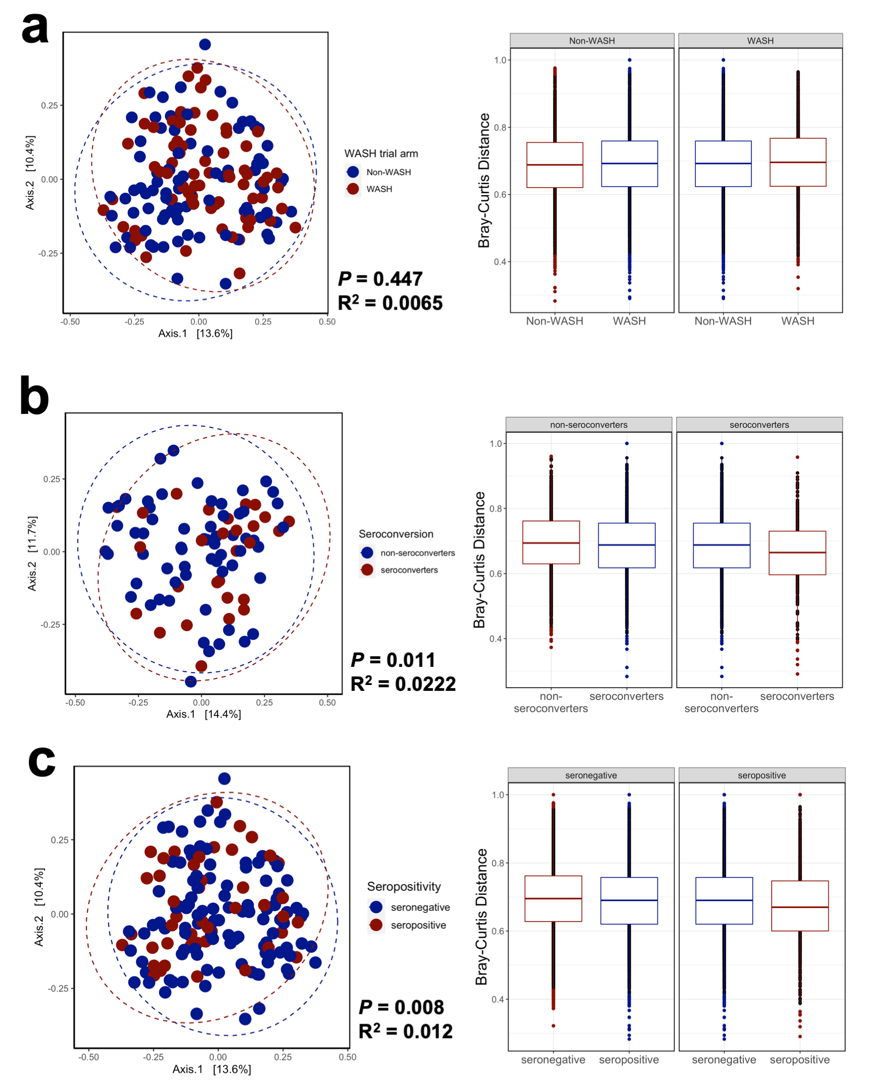


**Fig. S1.** PCoA and associated Bray-Curtis distances to assess beta diversity by randomized WASH trial arm (a), by seroconversion status in a restricted dataset including samples collected within 14 days of either vaccine dose (b) and by seropositivity status (c) using PERMANOVA analysis.


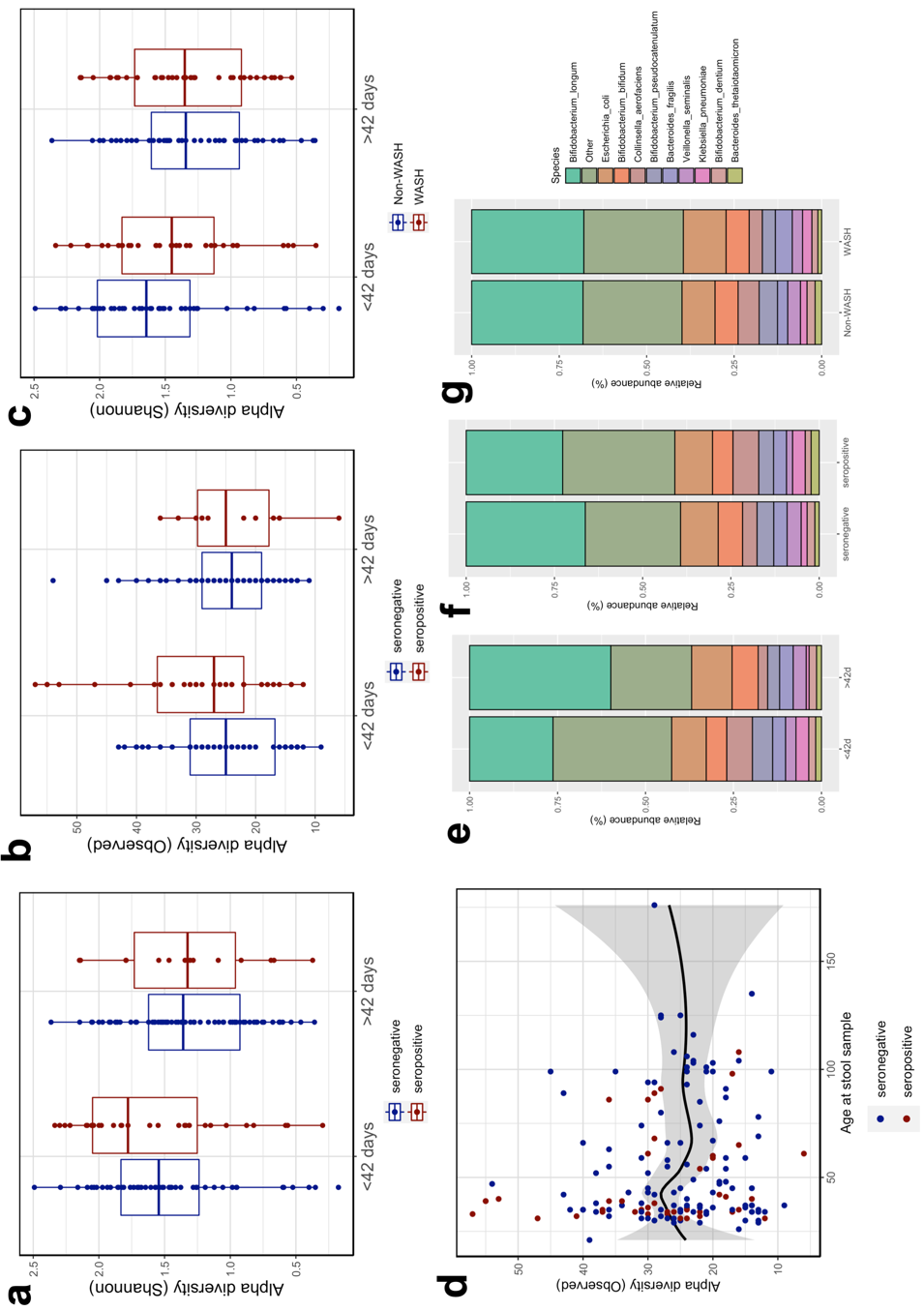


**Fig. S2.** Alpha diversity, as assessed using the Shannon index and number of observed species, between seropositive and seronegative infants (a-b) and by randomized WASH arm (c). (d) Associations between number of observed species and age at stool sample collection. Species composition in early vs late samples (e), seropositive vs seronegative infants (f), and WASH vs non-WASH infants (g).


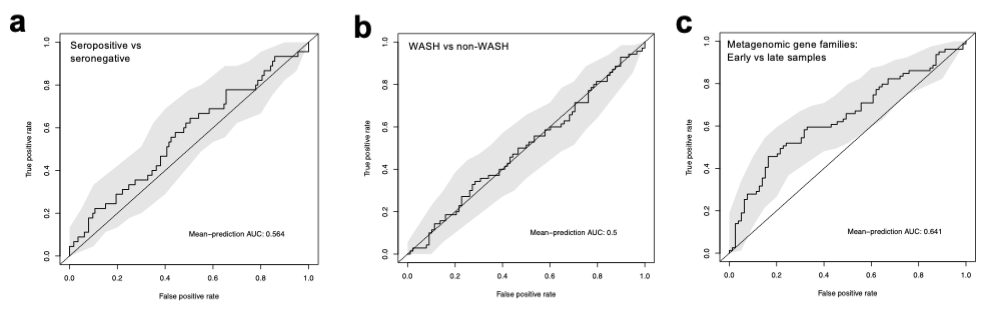


**Fig. S3.** Random forest classification ROC of models using taxonomic data to compare seropositivity status (a) and randomized WASH arm (b), and using metagenomic pathways to compare early vs late samples (c).


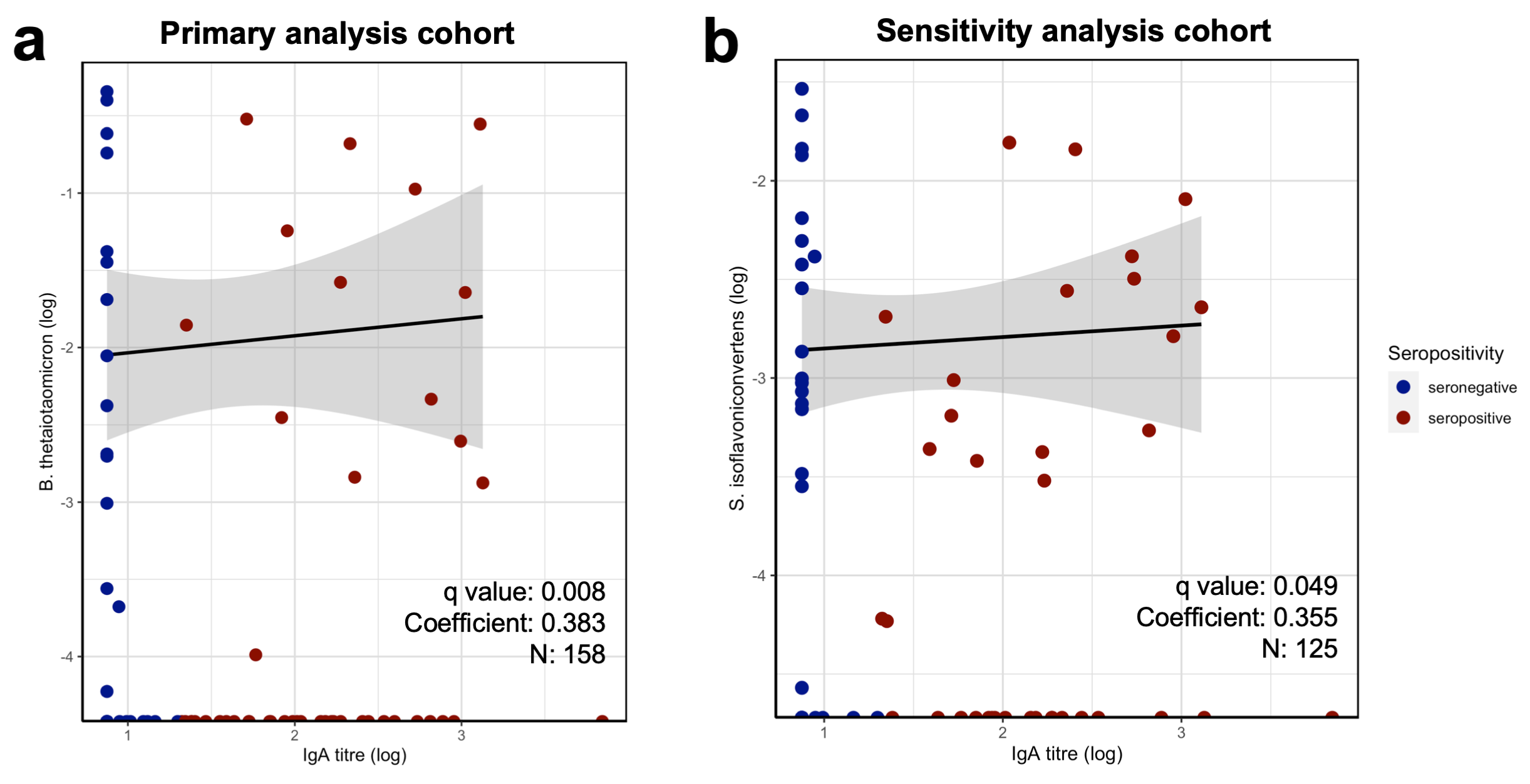


**Fig. S4.** Significant associations identified between RV IgA titre and the relative abundance of *B. thetaiotaomicron* (a) in the primary cohort and *S. isoflavoniconvertens* (b) in a sensitivity cohort using samples collected within 14 days of RVV. MaAsLin2 regression analysis (FDR corrected p value < 0.05).

**Table S1.**

|  | **SHINE study** | **RVV microbiome sub-cohort** |
| --- | --- | --- |
|  | **N=3831** | **N=158** |
| **Female, %** | 49.6 | 43.7 |
| **Birthweight, kg (sd)** | 3.08 (0.50) | 3.10 (0.50) |
| **Low birthweight, %** | 9.16 | 8.33 |
| **Vaginal delivery, %** | 92.4 | 95.6 |
| **Institutional delivery, %** | 88.8 | 94.3 |
| **Exclusively breastfed (at month 3), %** | 88 | 91.6 |
| **LAZ at 1 month visit (sd)** | -0.86 (1.35) | -0.92 (1.23) |
| **LAZ at 3 month visit (sd)** | -0.86 (1.34) | -0.92 (1.16) |
| **SHINE trial arm, %** |  |  |
| ***SOC*** | 24 | 26.6 |
| ***IYCF*** | 23.9 | 29.1 |
| ***WASH*** | 25.1 | 20.9 |
| ***WASH+IYCF*** | 27 | 23.4 |
| **Household size, median [IQR]** | 5 [3; 6] | 5 [4; 6] |
| **Mother age at enrolment** | 25.6 (6.59) | 27.5 (6.10) |
| **Mother height** | 160 (5.88) | 161 (5.98) |
| **Parity, median [IQR]** | 2 [1; 3] | 2 [1; 3] |
| **Mother mid upper arm circumference, cm (sd)** | 26.4 (3.10) | 27.4 (3.25) |
| **Unemployed, %** | 91.5 | 91 |
| **Open defecation, %** | 43.8 | 47.7 |
| **Any latrine, %** | 40.4 | 39.3 |
| **Improved latrine, %** | 35.6 | 36.7 |
| **Improved flood, %** | 55.3 | 57.8 |
| **Owns chickens, %** | 79.6 | 82.6 |
| **Livestock observed in house, %** | 36.6 | 48.1 |
